# Acute Erythroid Leukemia Post-Chemo-Radiotherapy and Autologous Stem Cell Transplantation Due to Multiple Myeloma: Tracing the Paths to Leukemic Transformation

**DOI:** 10.3390/ijms25148003

**Published:** 2024-07-22

**Authors:** Gábor Méhes, Attila Mokánszki, Anikó Ujfalusi, Zsuzsa Hevessy, Zsófia Miltényi, Lajos Gergely, Judit Bedekovics

**Affiliations:** 1 Department of Pathology, Faculty of General Medicine, University of Debrecen, 4032 Debrecen, Hungary; attila.mokanszki@med.unideb.hu (A.M.); bedekovics.judit@med.unideb.hu (J.B.); 2 Department of Laboratory Medicine, Faculty of General Medicine, University of Debrecen, 4032 Debrecen, Hungary; ujfalusi.aniko@med.unideb.hu (A.U.); hevessy@med.unideb.hu (Z.H.); 3 Department of Hematology, Institute of Internal Medicine, Faculty of General Medicine, University of Debrecen, 4032 Debrecen, Hungary; miltenyi.zsofia@med.unideb.hu (Z.M.); lgergely@med.unideb.hu (L.G.)

**Keywords:** erythroid leukemia, post-treatment leukemia, stem cell therapy, TP53 mutation, DNA-sequencing, melphalan treatment

## Abstract

The clinical impact of therapy-related acute leukemias is increasing with the extension of cancer-related survival; however, the origins remain largely unknown. Acute erythroleukemia (AEL), a rare unfavorable type of myeloid neoplasia, may also develop secondary to cytotoxic therapy. The disorder is featured by specific genetic alterations, most importantly multi-allelic mutations of the *TP53* gene. While AEL might appear as a part of the therapy-related MDS/AML, spectrum information regarding the genetic complexity and progression is largely missing. We present two AEL cases arising after cytotoxic therapy and melphalan-based myeloablation/autologous peripheral stem cell transplantation due to multiple myeloma (MM). As stated, multiple pathogenic *TP53* variants were present unrelated to preexisting MM, in parallel with uninvolved/wild-type hemopoiesis. Potential mechanisms of leukemic transformation are discussed, which include (1) preexisting preneoplastic hemopoietic stem cells (HSC) serving as the common origin for both MM and AEL, (2) the generation and intramedullary survival of p53-deficient post-chemotherapy HSCs, (3) reinoculation of mobilized autologous *TP53* mutated HSCs, and (4) melphalan treatment-related late-onset myelodysplasia/leukemia with newly acquired *TP53* mutations.

## 1. Introduction

With the prolonged survival of cancer patients, the treatment-related late complications become more and more obvious. The long-term effects of chemo-radiotherapy in both the hemopoietic stem cell pool and the bone marrow microenvironment (niche) culminate as leukemogenic stimuli, the significance of which is increasingly considered in the clinic. Myeloid neoplasia post-cytotoxic therapy (MN-pCT) is a major representant of secondary myeloid neoplasias, which evolve following the exposure to anti-cancer cytotoxic or irradiation therapy. Both the latest (the fifth) edition of the World Health Organization Classification of Hematolymphoid Tumors [1] and the International Consensus Classification of Myeloid Neoplasms [2] clearly recognize the initiating effect of prior genotoxic anticancer therapies in myeloid oncogenesis. As such, this etiology is supposed to be marked with the long-term persistence of subclinical preleukemic clones, befitting the definition of clonal hemopoiesis.

In addition to patients’ medical history, MN-pCT is determined by distinctive molecular biological/genetic features. This includes a complex karyotype with the frequent involvement of the chromosome 17p (*TP53*) locus, as well the exclusion of most of the somatic genetic abnormalities representing other well-defined molecular AML subgroups [3]. Alterations of the *TP53* gene, frequently in a bi- or multi-allelic form, result in high-risk disease with generally poor survival [4,5].

As a special form of myeloid neoplasia, acute erythroid leukemia (AEL) presents with aborted erythroid differentiation, a maturation block resulting in large proportions of proerythroblasts/pronormoblasts in the bone marrow. AML transforming from MDS with characteristic defects in the erythroid differentiation should also be considered as AEL, according to the current view. A genetic hallmark of AEL is the high prevalence of *TP53* mutations in a multiple/bi-allelic fashion, associated with aggressive behavior and therapy resistance.

While MN-pCT is an established disease category with a well-known etiology, AEL is infrequent and less intensively studied. Accordingly, the effect of therapeutic modalities is difficult to unravel in specific situations such as in patients treated for multiple myeloma (MM). To illustrate the pathological complexity, two cases of myeloid neoplasia post-chemotherapy are presented that completely fit in the category of AEL according to the WHO 2022 classification. Both disorders developed within 2.5 years (28 months and 13 months) after radio-chemotherapy and melphalan-based myeloablation followed by autologous peripheral stem cell transplantation, which proved to be effective to eradicate preexisting multiple myeloma. Our aim was to investigate and compare in detail the genetic and molecular features of the primary MM and of the secondary AEL as a unique combination and, further, to trace the potential origin of the leukemogenic clones from which all available samples from the two cases were analyzed.

### 1.1. Case 1

The 71-year-old male patient (NP) was introduced to the hospital with back pain in March 2019. Previous clinical anamnesis was limited to treatments due to rheumatological symptoms. Thoracic imaging presented a destructive/lytic lesion of the right thoracic paravertebral region; transthoracic needle biopsy resulted in a solid mass of plasmocytic neoplasia expressing CD138, CD56, and monoclonal Kappa light chain. Bone marrow flow cytometry reported 51% neoplastic plasma cells with CD38+/CD138+/CD56+/intracytoplasmic Kappa light chain immunophenotype, serum kappa light chain was elevated (941 mg/L), and serum electrophoresis also detected IgG-Kappa monoclonal M component (3.5 g/L). A genetic analysis of enriched bone marrow plasmocytes reflected chromosome 17 trisomy without signs of 17p loss. A hyperdiploid karyotype was confirmed by FISH analysis (four *FGF3* signals, three *CCND1* signals and three *MAF* signals; all FISH probes were from MetaSystems GmbH, Altlussheim, Germany; 200 cells were counted). Thus, ISS stage 2 myeloma with bone destruction was diagnosed. Clinical and morphological signs directing to myelodysplasia were not seen or reported by any of the investigations.

First-line fractionated irradiation (2 Gys; 40 Gys in total) of the thoracic vertebral mass was followed by a VTD (bortezomib, thalidomide, dexamethasone) chemotherapy combination (five cycles). Peripheral stem cells were collected with 3 g/m^2^ cyclophosphamide mobilization including G-CSF. Seven months after the primary diagnosis, the patient reached a very good partial response (VGPR) and autologous peripheral stem cell transplantation (APSCT) was performed with 3.55 × 10^6^/kg CD34+ cells infused after conventional melphalan conditioning (140 mg/m^2^). Engraftment was normal at day +9 after transplantation. The post-transplant controls indicated the recovery of bone marrow function with minimal M component in the serum, indicating MRD + CR. No post-transplant maintenance therapy was provided.

Thirty-five months after the primary diagnosis of multiple myeloma (August 2022) and twenty-eight months after melphalan-based myeloablation/APSCT, severe pancytopenia developed. Bone marrow flow cytometry displayed dysplastic hemopoiesis with massive erythroid hyperplasia of 44% immature erythroblasts and only 4.5% myeloblasts featuring CD34+/CD117+/CD33+/partialCD7+ phenotype. Plasmocytes were present in 0.1%, with likely aberrant CD138+/CD56+ phenotype in 0.01%, but the original clonality could not be demonstrated by high-sensitivity flow cytometry.

**Figure 1:**
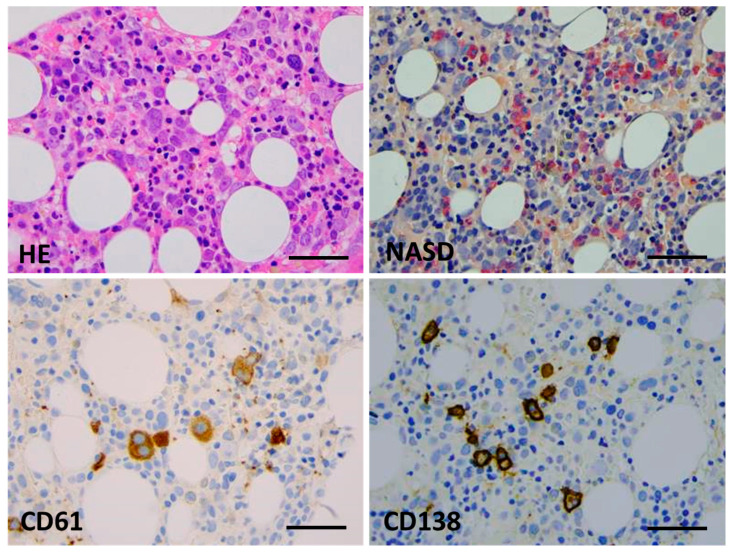
Bone marrow biopsy morphology (Case 1). Key features are hypercellular parenchyma with maturation defect and up to 80% of early erythroid precursors by conventional HE staining (**top left**), suppression of the myeloid lineage and a lack of terminal granulopoieses (in red, NASD histochemistry, (**top right**)), dysplastic megakaryocytes ((**bottom left**), CD61 IHC), and a few mature plasmocytes ((**bottom right**), CD138 IHC) (×400 virtual magnification, scale bar = 100 µm).

**Figure 2:**
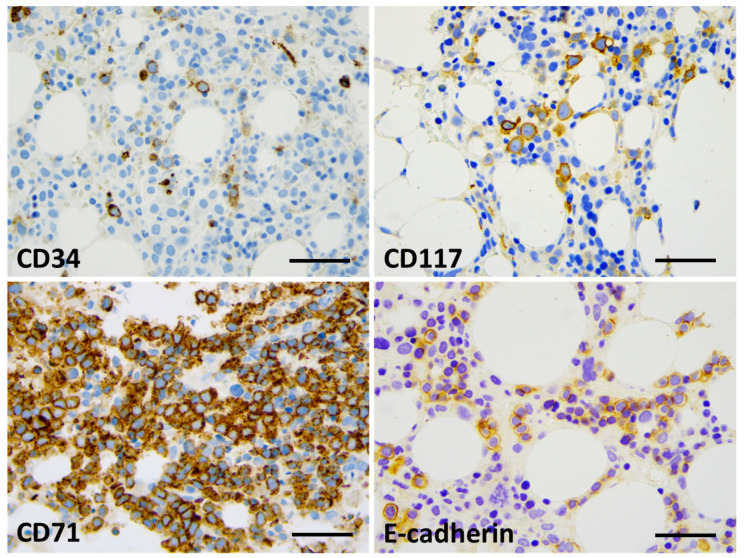
Bone marrow IHC showing limited CD34 labeling (up to 5%, with blast morphology) (**top left**) and approximately 15% CD117+ cells, mostly with proerythroblast morphology (**top right**). The great majority of cells (80%) presented with CD71 expression (**bottom left**); up to 30% of the cells presented with E-cadherin positivity and proerythroblast morphology (**bottom right**) (×400 virtual magnification, scale bar = 100 µm).

**Figure 3:**
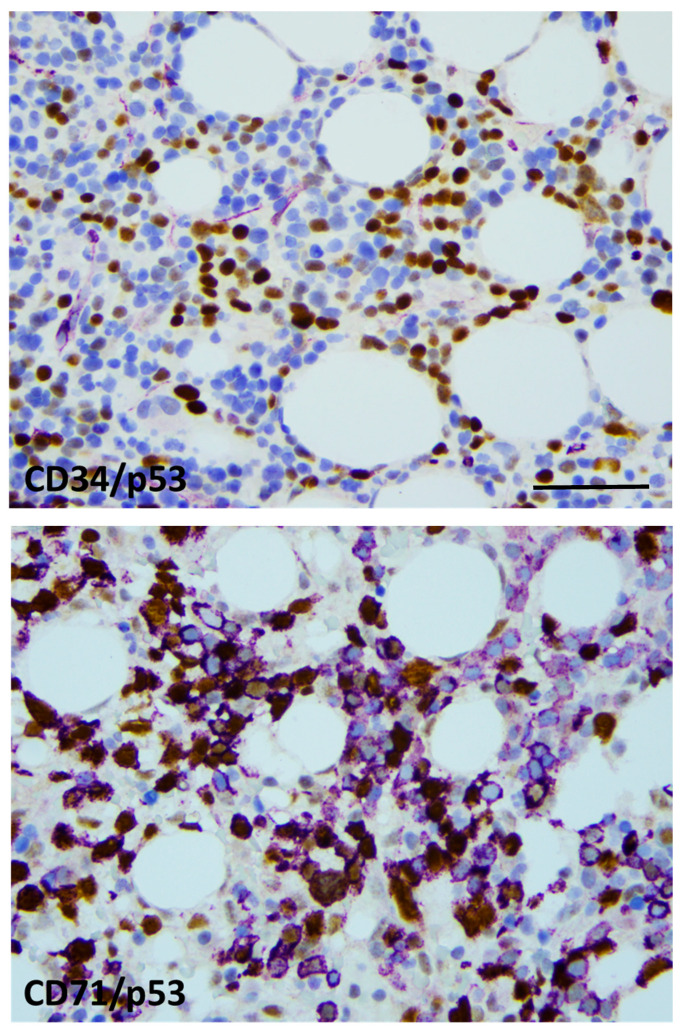
p53 labeling was restricted to the erythroid lineage. CD34/p53 double-IHC presenting generally with CD34 (violet)-negative and p53-positive (brown) blast cells (**top**), CD71/p53 double-IHC displaying CD71+ (violet) erythroblasts with and without p53 labeling (brown) (**bottom**). Strong nuclear p53 positivity refers to mutant *TP53* status in approximately 50% of the erythroblasts (×400 virtual magnification, scale bar = 100 µm).

Bone marrow biopsy morphology showed massive erythroid proliferation (80%) with large clusters megaloblasts (Figure 1). The myeloid lineage was strongly suppressed (myeloid/erythroid ratio of 1:5), like megakaryopoiesis. Dysplastic features dominated in the few residual megakaryocytes. CD138+ plasmocytes were seen in only 1–2%, with clustering around the BM sinuses, without obvious light-chain restriction (Kappa/Lambda = 1.0:1.5). The CD34+ fraction was below 3% while CD117+ marked early erythroid clusters. CD71+/E-cadherin+ immature erythroblasts occupied approximately 50% of the intertrabecular spaces (Figure 2). p53 immunohistochemistry using the antibody clone DO-7 (Dako-Agilent, Glostrup, Denmark) presented a generally strong nuclear reaction in approximately 50% of the bone marrow cells, consistent with a mutant *TP53* gene status. p53 immunopositivity dominated in the CD71+ erythroblasts fraction, as stated by CD71/p53 double IHC reactions (Figure 3).

**Figure 4:**
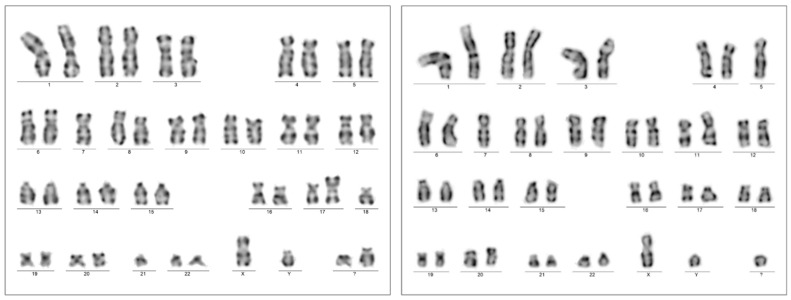
Chromosome karyotyping presented individual subclones with two unrelated chromosome 17p alterations from the same bone marrow sample with AEL diagnosis (Case 1). Subclone 1 was highlighted by the karyotype 45,XY,-7,add(17)(p13.?3),-18,-21,+2mar[5] (**left**); one of the marker chromosomes is a potential derivate of chromosome 18 (not further analyzed), in contrast to subclone 2, with the karyotype 45,XY,-5,-7,add(11)(p15),del(17)(p13.?1),+mar[3] (**right**). A significant portion of dividing cells presented with the normal karyotype 46,XY[12].

A chromosome analysis of the bone marrow aspirate resulted in a complex karyotype with bi-clonal presentation 45,XY,-7,add(17)(p13.?3),-18,-21,+2mar[5]/45,XY,-5,-7,add(11)(p15),del(17)(p13,?1)+mar[3]/46,XY[12].nuc ish(TP53x1,D17Z1x2)[50/200] (Figure 4). A FISH analysis of the 17p chromosome region (MetaCyte p53 FISH probe, MetaSystems, Altlussheim, Germany) was conducted on unsorted bone marrow cells and the loss of one of the p53 signals was found in 25% of the evaluated nuclei (50/200).

NGS sequencing of the *TP53* gene covering exon 1 to 11 hotspots (comprehensive p53 panel, Swift BioScience, Ann Arbor, MI, USA) was performed from DNA isolated from whole bone marrow material. Altogether, three *TP53* variants were detected at *TP53 c.713G>A*; *p.Cys238Tyr* (VAF: 44.26%), *TP53 c.422G>A*; *p.Cys141Tyr* (VAF: 11.07%) and *TP53 c.761T>A*; *p.Ile254Asn* (VAF: 10.64%). All variants were classified as pathogenic (Tier I) according to the COSMIC and the ClinVar databases [6]. No further variants were found using the 75 gene VariantPlex Myeloid NGS Panel (Archer, Boulder, CO, USA).

Based on the patient history of chemo-radiotherapy and APSCT, plus the recent findings of neoplastic erythroid proliferation featuring TP53 alteration, the diagnosis of acute erythroid leukemia post-cytostatic therapy was made, in agreement with the fifth edition of the WHO classification.

Despite the intensive treatment (azacytidine + venetoclax), the general condition gradually declined and the patient died 4 months after the AEL diagnosis at the age of 74 years. Autopsy findings stated the bone marrow involvement of over 30% of CD71+ erythroblasts; no residual myeloma could be stated.

### 1.2. Case 2

The 51-year-old male patient (LT) presented with progressive paraparesis due to an infiltrating tumor at the level of the thoracic VIII in January 2017. The decompression and subtotal removal of the tumor mass was carried out in an acute setting. Histology and laboratory findings identified a plasmocytic multiple myeloma, lambda clonal, with 15% plasmocytic infiltrate of the bone marrow. Cytogenetics resulted in monosomy 13 in 13% of the evaluated cells. Clinical ISS stage 1, with bone lesion and anemia, was noted. Fractionated irradiation (20 Gy) was applied within 1 month, followed by four cycles of VTD combination over a period of four months. Peripheral stem cells were collected after mobilization according to the cyclophosphamide/G-CSF protocol. Autologous peripheral stem cell transplantation was performed after melphalan (200 mg/m^2^) conditioning, 52 months after the initial diagnosis (July 2021), without significant unexpected events. The patient remained in complete remission for over a year.

**Figure 5:**
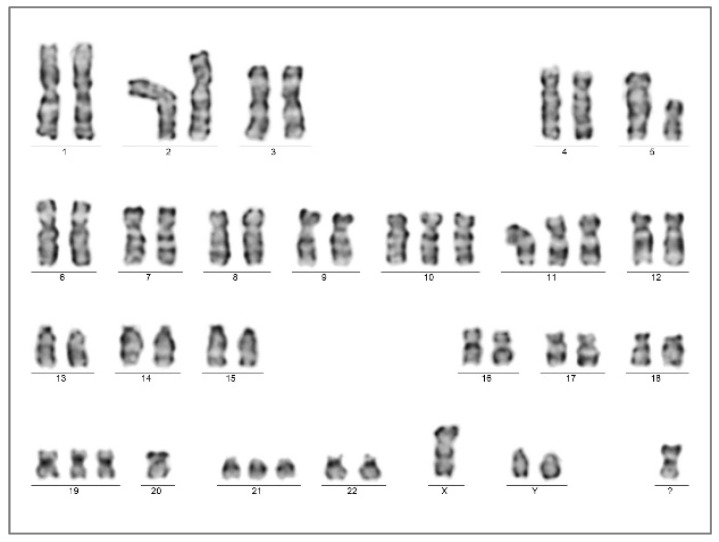
Bone marrow chromosome analysis presented the complex karyotype of 51,XY,+Y,del(5)(q12q3?5),+10,+11,+19,-20,+21,+mar[20] at the time of the AEL diagnosis (Case 2).

The patient presented with weakness and anemia/pancytopenia from August 2022, 13 months after myeloablation/APSCT. BM examination identified hypercellularity (80%) with megaloblastic and dysplastic morphology. CD34+/CD117+ blast counts raised from 6% to 15% within a month, while a massive proliferation of early CD71+/E-cadherin+ erythroblasts with megaloblast cytomorphology, comprising up to 60%, were consistently observed. Strong p53 IHC positivity was limited to CD71+ erythroblasts. Bone marrow genetics resulted in the complex karyotype of 51,XY,+Y,del(5)(q12q3?5),+10,+11,+19,-20,+21,+mar[20] without a visible change at chromosome arm 17p (Figure 5).

The laboratory findings first suggested the clinical diagnosis of AML-pCT, IPSS-R:10 with unfavorable cytogenetics. Non-clonal CD138+ plasmocytes were present in the bone marrow at a rate of 10% (Kappa/Lambda = 1:1).

NGS directed to the *TP53* gene alterations presented two variants with different allele frequencies: *c455C>T*; *p.Pro152Leu* (VAF 38.69%) and *c.659A>G*; *p.Tyr220Cys* (VAF 6.28%). Repeated analysis after 4 months resulted in highly similar values (VAF 33.0% and 4.7%, respectively). No further pathogenic variants were detected using the myeloid NGS gene panel.

Based on the mass of erythroblasts with a complex karyotype and *TP53* mutations, the diagnosis of MDS/AML-AEL post-chemotherapy was finally made. Combined therapy of azacytidine + magrolimab (seven cycles) followed by the 7 + 3 protocol plus venetoclax was applied, with massive supportation due to severe anemic and septic periods (last presentation in August 2023).

## 2. Discussion

The expansion of immature erythroid precursors was first described by Di Gulielmo as erythremic myelosis in 1917, and thus this was referred as Di Guglielmo’s disease for decades [7]. Since then, the diagnosis of erythroid MDS/erythroleukemia has been based on objective criteria, including dysplastic features, blast counts, and the mass of immature erythroids. In the 2008 WHO Classification of Hematological Neoplasias, acute erythroid leukemia was split into two subtypes—erythroleukemia and pure erythroid leukemia (PEL)—whereas in the 2016 WHO update, erythroleukemia was merged into myelodysplastic syndrome and PEL was renamed as acute erythroid leukemia [8]. AEL is precisely defined and was also integrated in the 2022 classifications of myeloid neoplasias (both WHO and ICC). Dysplastic morphology is common for both MDS and erythroleukemia; thus, AEL most likely represents disease progression from a preexisting MDS. In line with this, the genetic abnormalities associated with erythroleukemia are closer to those seen in MDS than to those of de novo AML, as also reflected by the common *TP53* mutations and the absence of *FLT3* and *NPM1* mutations [9]. Within the subset of erythroleukemia, a direct crosstalk between *Gata1* and *TP53* has been described, suggesting a lineage-restricted transforming activity [10].

AEL is a rare and aggressive form of acute leukemia. By definition, the following two criteria should be met: (1) erythroid cells usually comprising ≥80% of total nucleated bone marrow cells, (2) of which ≥30% are erythroblasts with maturation arrest. Erythroid differentiation is highlighted by CD71 (transferrin receptor), E-cadherin, and glycophorin-A expression; partial CD117 and CD33 expression is permitted, while CD34 and HLA-DR remain mostly negative [11,12,13]. The challenging aspect at presentation is the reverse pattern of erythroid proliferation and myelosuppression, which calls for definitive clonal genetic/biological features. At the cytogenetic level, AEL usually has a complex karyotype, with frequent abnormalities involving chromosomes 5 and 7 [14]. Occasional fusion genes secondary to chromosomal translocations have also been described [15,16]. As a characteristic feature, AEL is associated with the involvement of chromosome 17p, harboring the *TP53* gene [17]. *TP53* loss and/or somatic mutations of the gene occur in approx. 70% of cases, frequently with a bi-allelic status [18]. The experimental data indicate that the occurrence of *TP53*, and further *BCOR*, *DNMT3A*, *RB1*, and *NFIX* mutations, may contribute to the development of leukemia with an erythroid phenotype [19,20].

There are limited data available on the development of secondary AEL in association with post-treatment conditions. Tashakori et al. studied 25 patients with AEL/PEL, including 16 de novo and 9 therapy-related cases. Both groups had comparable clinical findings and overall survival [21]. *TP53* mutations, commonly missense variants, were present in 10/16 (62.5%) of the de novo group and in 8/9 (89%) cases of the therapy-related group. Notably, monosomy 17 or del(17p) were more common in the therapy-related group (71.4% vs. 26.6%), underscoring the significance of distinctive *TP53* alterations, possibly reflecting improved survival and a fitness advantage. In another study, Fang et al. also focused on therapy-related AEL (*n* = 11), all featuring *TP53* mutations with the involvement of the DNA-binding domain in 88% (exons 5–8, median VAF 35%). Additional gene mutations were identified in the minority of cases, as follows: *DNMT3A* (*n* = 3; VAF 10.3–29.3%), *NRAS* (*n* = 2; VAF 5% and 26.6%), *TET2* (*n* = 1; VAF < 3%), *FLT3* (*n* = 1; VAF 1.7%), *PRPF40B* (*n* = 1; VAF 40.8%), *KMT2A* (*n* = 1; VAF 15.2%), and *GATA2* (*n* = 1; VAF 1.8%) [22].

Therapy-related myeloid neoplasms in patients with myeloma were also repeatedly studied in the past decade. Secondary MDS/AML could be observed in 2.1% of patients treated for MM. In addition, the synchronous presentation of MDS/AML was also identified in a minor subset (0.53%), indicating a low but real risk of preexisting preleukemic status that should be carefully excluded before adducing therapy-related mutagenesis [23].

For a while, frontline or delayed melphalan-based autologous stem cell transplantation was a standard treatment, a regimen achieving excellent response rates [24]. Unfortunately, high-dose melphalan (HDM) was also reported as a potent genotoxic agent [25,26]. The association of HDM with myeloma cell transformation and late remission of the disease has been established. Moreover, its adverse effect on hemopoietic stem cells by inducing secondary MDS/AML also became a matter of investigation. In a recent study evaluating 66 melphalan-treated cases, the biological characteristics were found to be consistent with the usual pCT group of myeloid malignancies presenting with a complex karyotype (48.5%) and *TP53* mutations (67.2%), with the latter being the sole mutation in over one third of cases. Unfortunately, information about specific myeloid WHO categories is not included here; thus, the frequency of AEL remains hidden. [27].

Going back to the mid-1970s, AEL was sporadically reported in association with MM [28,29,30,31]. More recent case presentations demonstrate standard immunophenotypic and genetic features, including chromosome 17 aberrations [32,33]. Our current cases represent the most precise clinic-pathological and genetic description to date. The cytogenetically complex heterogenous leukemic pool presented with two/three different pathogenic *TP53* variants, indicating the multi-allelic involvement of the gene. Based on the *TP53* variants collected from the NGS files, a dominant clone is represented (VAF 44.26% and 38.69%, respectively), with an additional one or two minor subclones featuring secondary *TP53* variants with constantly lower VAF values (VAF 6.28–11.07%). Interestingly, repeated chromosome analysis also indicated that a significant proportion (approx. 50%) of functionally active (dividing) bone marrow cells have normal karyotype and no visible 17p alteration in both cases.

**Figure 6:**
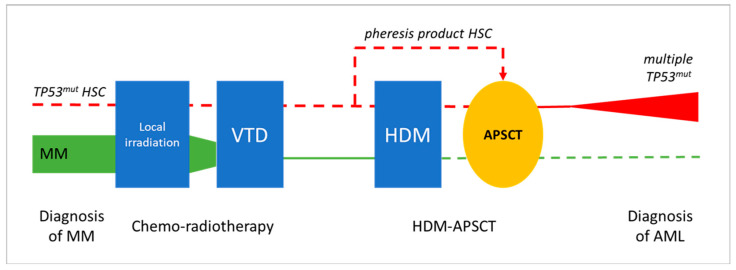
Potential evolution leukemic transformation and the development of pCT AEL following HDM-APSCT due MM. Chemo-radiotherapy and delayed APSCT were effective in treating MM in both presented cases (green line illustrates declining myeloma tumor burden). Secondary AEL showing characteristic *TP53* mutations in immature erythroblasts as a unique feature could be the result of the transforming effect of VTD chemotherapy, with or without residual mutant HSCs, or of the HDM therapy (red line). Clonal aberrations in prior treatments, as well as in the pheresis product, could not be observed via NGS; thus, HDM-induced genotoxicity is favored as the most likely mechanism of leukemogenesis. (symbolic appearance of preleukemic/leukemic TP53 mutated myeloid clone in red, disappearance of the myeloma clone in green, dashed line represents lack of evident involvement in the presented case studies).

In the presented cases, AEL developed within 2.5 years following combined CT and HDM-APSCT, representing a special post-treatment situation. At the frontline, MM was treated by the widely used VTD chemotherapy regimen and showed an excellent long-term response in both cases. Peripheral blood hemopoietic stem cells were collected during complete remission of the disease and delayed HDM-based PSCT was successfully applied, leading to the long-term remission of MM. The development of two secondary AEL with similar clonal/subclonal changes and patient histories suggests a direct association with prior cytotoxic therapy. The exact mechanism and timing of stem cell transformation and leukemic progression is difficult to address. The following potential options could be considered (see Figure 6).

(1) Preexisting/synchronous clonal hemopoiesis serves as initiating event for both MM and AEL. Nothing really supports a common origin of the two hematological diseases; primary myeloma bone marrow did not show myelodysplastic or proliferative features. Moreover, MM cells were free of 17p and *TP53* alterations.
(2) The occurrence of preleukemic hemopoietic stem cell (HSC) transformation due to front-line chemo-radiotherapy (VTD combination), as they locally survive the myeloablative treatment. If transformed HSC clones are highlighted by *TP53* mutation, they are supposed to gain resistance to the applied treatment. According to relevant findings, pre-leukemic HSCs with clonal changes have a growth advantage over non-mutated HSCs [34,35]. In addition, bone marrow stromal niches may supply relative protection and thus also contribute to survival, despite myeloablative therapy.
(3) The mobilization, harvesting, and re-inoculation of clonal/preleukemic HSCs after myeloablative treatment. The unwanted collection or even enrichment of a *TP53* mutant “pre-leukemic” stem cell fraction following mobilization would be possible. To prove this opportunity exists, our archives were checked for remains of HSC pheresis products. Unfortunately, five years after the procedure, nothing was available for Case 1. However, the pheresis product of Case 2 could be tested for TP53, although it did not show any alterations despite the high amount and good quality of the DNA obtained from the frozen sample. Therefore, in at least one of the demonstrated cases, we could exclude the direct transfer of *TP53*-mutant preleukemic cells by the pheresis product collected before the high-dose melphalan hit.
(4) HDM-induced mutagenesis. According to this option, melphalan genotoxicity results in *TP53* mutant pre-leukemic clones rising from the residual HSCs of an otherwise normal, myeloma-free, bone marrow. Mutant HSC clones do not show a proliferation advantage per se and progression is expected in a slow fashion, with HSC clones remaining uncovered for longer times. The severe dysplasia, complex karyotype, and multiple *TP53* mutations perfectly fit the features of the melphalan-related biological signature [25,26]. The exclusion of prior signs of leukemia and the presence of typical clinical–biological features favor HDM as the most likely inducer of secondary myeloid neoplasia in both presented cases.

## 3. Conclusions

AEL is part of both the recent classifications of myeloid neoplasias. While the fifth WHO system includes the separate entity of post-cytostatic MDS/AML, the ICC mentions therapy-related conditions as diagnostic qualifiers to be applied following specific MDS or AML diagnostic classes. However, therapy-related conditions can be highly variable, and the potential complications of myeloablation/autologous stem cell transplantation are also at risk. As discussed, the survival of minor subclones, the potential oncogenic effects of the myeloablative therapy, or even the re-inoculation of preleukemic HSCs should be considered in the background of secondary MDS/AML in such cases. After the exclusion of all other options, HDM treatment should be preferred as the most likely inducer of leukemic transformation in the presented cases; however, an exact explanation of the uncommon AEL-type differentiation could not be provided.

The availability of high-resolution NGS-based genetic analysis and the demonstration of progressive leukemogenic changes, such as pathogenic *TP53* variants, makes the identification of therapy-related unfavorable biological factors and the long-term risks of secondary complications increasingly possible. Potential preleukemic alterations could therefore be captured much earlier by molecular monitoring in the exposed population.

## Data Availability

No publicly archived datasets available for this work.
